# Ectopic Sequestered Thyroid Tissue: An Unusual Cause of a Mediastinal Mass

**DOI:** 10.5402/2011/313626

**Published:** 2011-04-17

**Authors:** A. D. Mace, A. Taghi, S. Khalil, A. Sandison

**Affiliations:** ^1^Department of Otolaryngology, Charing Cross Hospital, Imperial College Healthcare NHS Trust, Fulham Palace Road, London W6 8RF, UK; ^2^Department of Histopathology, Charing Cross Hospital, Imperial College Healthcare NHS Trust, Fulham Palace Road, London W6 8RF, UK

## Abstract

An 80-year-old female presented with an incidental finding of a retrosternal mass on magnetic resonance imaging. Ultrasound demonstrated a mediastinal lesion adjacent to but separate from the inferior pole of the right thyroid lobe. Fine needle aspiration cytology demonstrated colloid and follicular cells. At surgery, the right thyroid lobe was found to be normal. A discrete 5 cm nodule was found in the anterior mediastinum separate from the thyroid and just anterior and to the right of the trachea and thymus. The nodule had a vascular pedicle arising from the mediastinum. The differential diagnosis included metastatic thyroid carcinoma. Histology was consistent with a benign ectopic sequestered thyroid nodule. Extensive investigations demonstrated no sign of a thyroid malignancy.

## 1. Introduction

Ectopic thyroid tissue is a common abnormality and results from abnormal embryologic development and migration of the thyroid gland. Such tissue is usually found along the path of descent of the thyroid gland in the anterior midline of the neck. A lingual thyroid is the most common presentation of thyroid ectopy [1] along with thyroglossal duct remnants [2]. Ectopic thyroid tissue has been described in other parts of the head and neck such as the submandibular [3, 4] and parotid salivary glands [5]. Ectopic thyroid tissue in other locations is rare. There are case descriptions of thyroid tissue identified in diverse locations such as the axilla [6], trachea [7–10], adrenal [11], small intestine [12], and porta hepatis [13]. The most frequent noncervical location for ectopic thyroid tissue is the thoracic cavity [14–18]. The differential diagnosis of abnormally located thyroid tissue must include metastatic thyroid carcinoma. Here, the authors describe a case of ectopic anterosuperior mediastinal thyroid mass excised through a cervical incision and the subsequent investigation to exclude malignancy. 

## 2. Case Report

**Figure 1:**
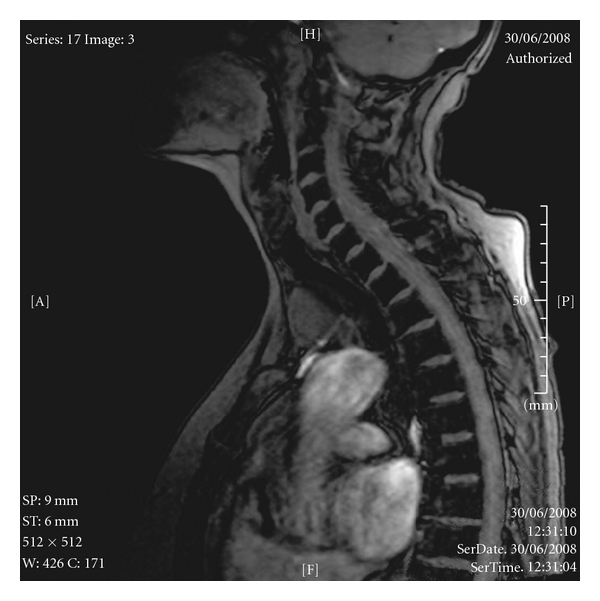
Lateral magnetic resonance image.

**Figure 2:**
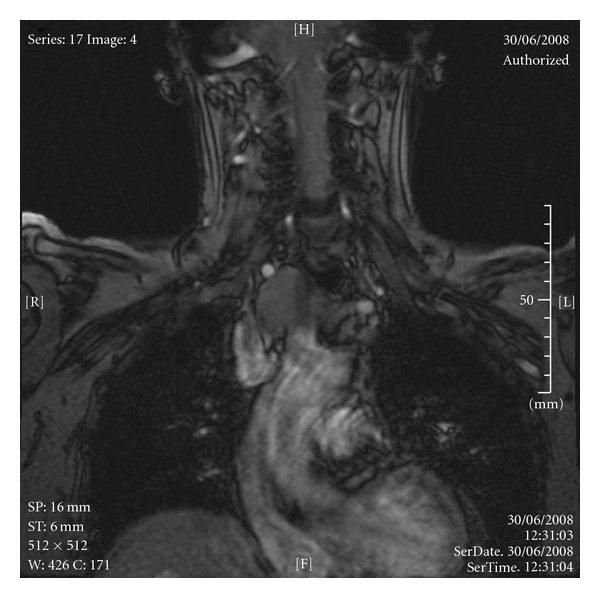
Anteroposterior magnetic resonance image.

An 80-year-old nonsmoker female patient presented with an incidental finding of a mediastinal mass on MRI scan for investigation of vertigo (Figures 1 and 2).

Her previous medical history included asthma, osteoporosis, and bilateral leg lymphoedema. Thyroid stimulating hormone (TSH) and free thyroxine (fT4) were within the normal range, and routine preoperative blood tests were normal.

**Figure 3:**
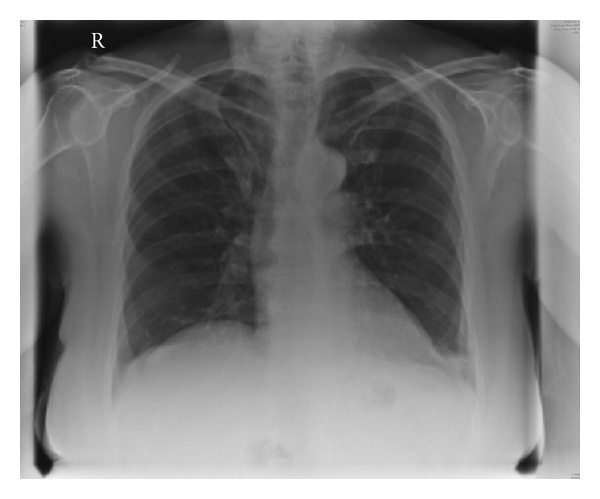
Anteroposterior chest radiograph demonstrating deviation of the trachea around right mediastinal mass.

**Figure 4:**
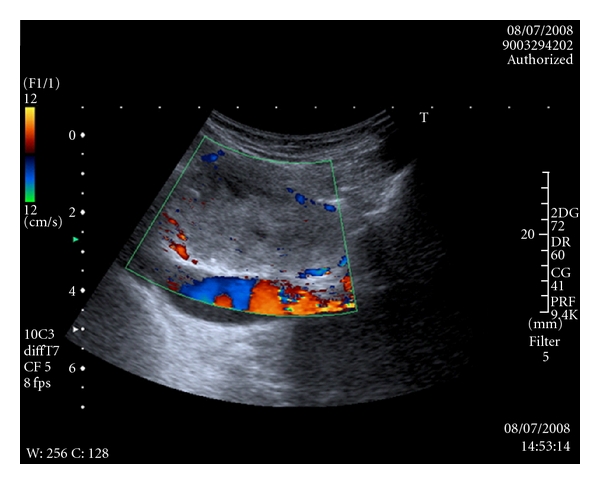
Colour flow Doppler ultrasound image of superior mediastinal mass.

An anteroposterior chest radiograph demonstrated deviation of the intrathoracic trachea around the mass (Figure 3). A subsequent ultrasound scan demonstrated a superior mediastinal mass separate from the inferior pole of the right thyroid lobe measuring 4.8 cm in maximal diameter (Figure 4). The mass was displacing the superior mediastinal vessels inferiorly. Ultrasound guided fine needle aspiration biopsy was performed on two occasions. The first sample was inadequate (THY1), the second sample demonstrated only colloid and a few follicular cells. Since malignancy could not be definitively ruled out, the mass was excised through a cervical incision. 

### 2.1. Operative Procedure

Under general anesthesia, the patient was placed in the supine position with the neck extended, prepared, and draped. Endotracheal laryngeal nerve monitoring was used with NIM contact EMG endotracheal tube and NIM-response 2.0 monitor (Medtronic USA, Inc.6743 Southpoint Drive North, Jacksonville, Florida, USA, 32216-0980).

Through a standard 5 cm midline cervical incision, the right thyroid lobe was dissected, found to be grossly normal, and excised. Both right parathyroid glands were found in the usual locations and preserved. The right recurrent laryngeal nerve was identified in the normal position, confirmed functioning with nerve stimulation at 2 mA, and preserved. Separate and inferior to the right thyroid lobe a discrete encapsulated mass was identified in the superior, mediastinum. This extended across the anterior surface of the trachea to the left side, adjacent to the thymus. The mass was separated from the thyroid gland by fat, in the same tissue plane but with no identifiable connection to the cervical region. The mass was excised through the cervical incision. During excision of the mass, a double vascular pedicle, arising from inferiorly in the mediastinum, was ligated. The patient had an uncomplicated postoperative recovery. Vocal cord function was normal on postoperative flexible endoscopy. 

### 2.2. Subsequent Investigations

**Figure 5:**
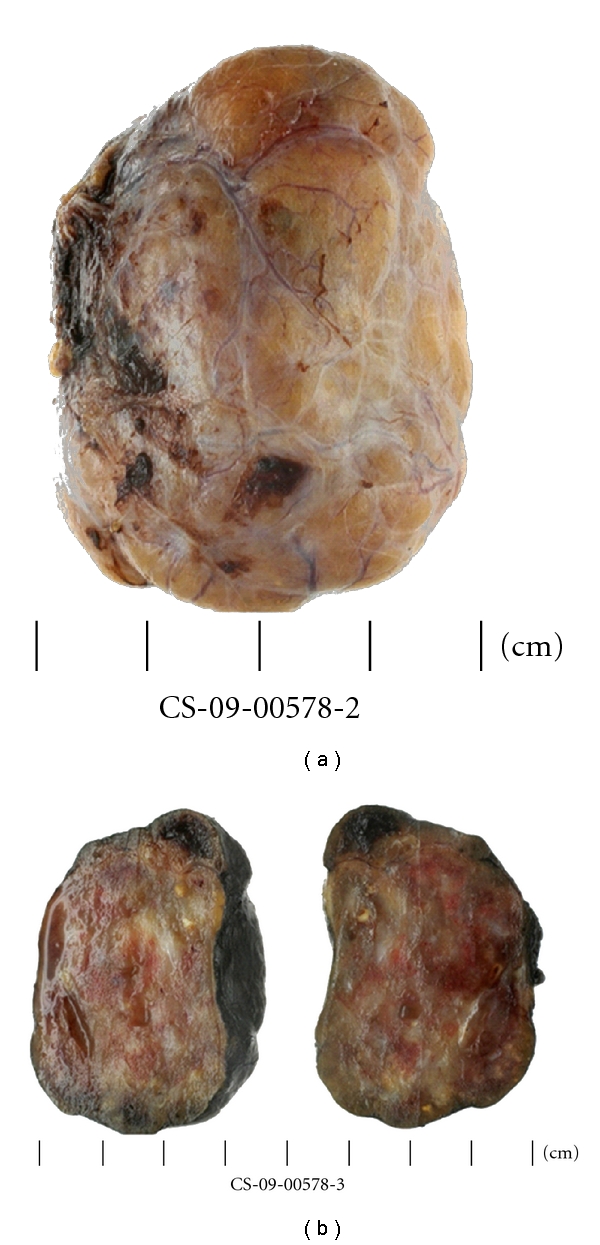
Macroscopic image of excised mediastinal thyroid mass (a) is the outer surface and (b) is the cut surface.

**Figure 6:**
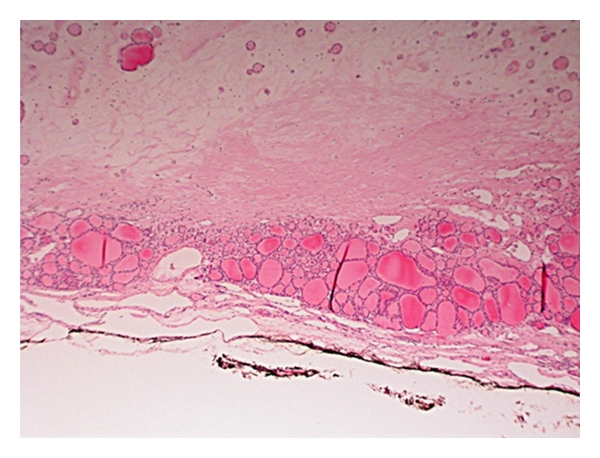
H&E stained section viewed at low power showing a peripheral rim of preserved thyroid follicles around a central paucicellular fibrotic area.

Histopathologic evaluation of the excised mediastinal specimen demonstrated a 5.3 cm by 2 cm thyroid tissue mass with large oedematous loose areas and foci of calcification and fibrosis (Figure 5). No neoplasia was seen (Figure 6). The excised right thyroid lobe had a nodular architecture composed of follicles of varying sizes with focal lymphoid aggregates.Thyroid function tests at 3 months were normal. Further investigation with whole body radioiodine I123 scintigraphy demonstrated expected residual uptake in the remaining left thyroid lobe and surgical bed but no uptake suggestive of other ectopic thyroid tissue or metastatic disease. Ultrasound surveillance of a 9 mm nodule in the remaining left lobe demonstrated no change in size over 6 months. 

## 3. Discussion

In humans, the thyroid gland begins to develop at the 24th day of gestation. The thyroid is the first endocrine gland to develop and originates from between the first and second branchial arches. An invagination of endodermal epithelial cells begins at the midline of the developing pharyngeal floor forming a diverticulum. This site, known as the foramen caecum, lies between the tuberculum impar (median tongue bud) and the hypobranchial eminence (copula). The foramen caecum can be observed in adults as a small pit at the base of the tongue where the tongue is divided into an oral anterior two-thirds and pharyngeal posterior third by the sulcus terminalis.

The initial path of descent of the bilobed thyroid diverticulum is anterior to the pharyngeal gut, the hyoid bone, and the laryngeal cartilages. At the same time that the primitive thyroid is migrating, the hyoid bone is developing. Embryological studies have shown that the strap muscles pull downward on the hyoid bone during development causing a forward tilt. This tilt pulls the tract posteriorly and causes it to be hooked up behind the hyoid bone. The tract of the developing thyroid is therefore closely associated with the hyoid bone. This explains the importance of excision of the median portion of the hyoid bone in the Sistrunk procedure to excise a thyroglossal duct cyst. 

The gland reaches its final location anterior to the trachea by the 7th week gestation. By this stage, it has acquired a median isthmus and two lateral lobes. As it descends, it is joined by the ultimobranchial bodies which form the parafollicular C cells.

Thyroid function begins at around the end of the 3rd month when the follicular cells commence production of colloid and follicles appear. 

During migration, from the 5th week gestation, the thyroid is still connected to the tongue by the thyroglossal duct. In normal development, this tubular structure subsequently obliterates entirely at approximately the 8 to 10th week. In some individuals, however, abnormalities in the embryologic development and migration of the thyroid gland can result in ectopic thyroid tissue. These remnants can appear at any point along the migratory path and are always located at or near the midline. The majority of remnants are found at, or just inferior to, the body of the hyoid bone as a thyroglossal cyst. Remnants can also be found in the tongue base as a lingual thyroid or close to the thyroid cartilage. The inferior end of the thyroglossal duct may fail to obliterate, and in at least half of individuals, a pyramidal lobe of the thyroid can be seen to persist. This pyramidal lobe itself may be attached to the hyoid bone or may be incorporated into a thyroglossal duct cyst.

Thyroglossal duct remnants may be asymptomatic but may cause locally obstructive or infective complications. Ectopic thyroid tissue is subject to the same diseases as the gland itself. Hypothyroidism is common in lingual thyroid, and thyroid supplementation is generally required. 

Ectopic thyroid tissue in other locations is rare and generally the subject of single case reports. There are a number of theories regarding the aetiology of such ectopic tissue. The most important diagnosis to exclude is metastatic lymph node deposits of well-differentiated thyroid carcinoma. Indeed, it is generally accepted that early reports of “lateral aberrant” thyroid tissue may represent papillary thyroid carcinoma metastases. Another hypothesis is that a thyroid nodule may become detached from the gland. In this case, the vascular supply should come from branches of the superior or inferior thyroid artery. There are reports of thyroid deposits in the soft tissues of the neck representing surgical implantation of thyroid neoplasms. In one case, infiltrating thyroid tissue in muscle and fibrous tissue presented 3 years after major blunt trauma to the neck. The tissue resembled that in a disrupted thyroid nodule present in the gland itself and was regarded as traumatically implanted [19]. There are, however, a number of ectopic thyroid masses reported which do not have features consistent with these theories and are considered truly developmental. Animal studies suggest that ectopic location of thyroid tissue may be related to vascular development [10].

The normal arterial supply of the thyroid gland consists of paired superior and inferior thyroid arteries. The superior thyroid artery is generally considered to be present in 100% of cases, and its absence has only been reported once [20]. An unusually large superior thyroid artery may replace the contralateral vessels or the inferior thyroid artery. The inferior thyroid artery has been reported as absent or doubled. The inferior thyroid artery when unusually small or absent may be replaced or supplemented by a thyroidea ima artery. Published reports place the incidence of a thyroidea ima artery between 2% and 12% [21]. 

In the case described here, there was no history of trauma or previous neck surgery. The ectopic mass was separate from the thyroid gland but in the same anatomical plane. The vascular supply clearly came from inferiorly within the mediastinum. Careful histological examination of the mass and the excised ipsilateral thyroid lobe demonstrated no evidence of neoplasia, and exhaustive investigations showed no evidence of thyroid malignancy elsewhere. The authors therefore conclude that this represents developmentally ectopic sequestered thyroid tissue in the anterior mediastinum. Despite this, the authors suggest that abnormally situated thyroid tissue, other than that in the central neck along the path of embryological descent of the thyroid gland, should be excised for careful histological analysis in order to exclude metastatic disease.
